# Dynamics enhanced by HCl doping triggers 60% Pauling entropy release at the ice XII–XIV transition

**DOI:** 10.1038/ncomms8349

**Published:** 2015-06-16

**Authors:** K. W. Köster, V. Fuentes-Landete, A. Raidt, M. Seidl, C. Gainaru, T. Loerting, R. Böhmer

**Affiliations:** 1 Fakultät Physik, Technische Universität Dortmund, D-44221 Dortmund, Germany; 2 Institute of Physical Chemistry, University of Innsbruck, A-6020 Innsbruck, Austria

## Abstract

The pressure–temperature phase diagram of ice displays a perplexing variety of structurally distinct phases. In the century-long history of scientific research on ice, the proton-ordered ice phases numbered XIII through XV were discovered only recently. Despite considerable effort, none of the transitions leading from the low-temperature ordered ices VIII, IX, XI, XIII, XIV and XV to their high-temperature disordered counterparts were experimentally found to display the full Pauling entropy. Here we report calorimetric measurements on suitably high-pressure-treated, hydrogen chloride-doped ice XIV that demonstrate at the maximum 60% of the Pauling entropy is released at the transition to ice XII. Dielectric spectroscopy on undoped and on variously doped ice XII crystals reveals that addition of hydrogen chloride, the agent triggering complete proton order in ice XIV, enhances the precursor dynamics strongest. These discoveries provide new insights into the puzzling observation that different dopants trigger the formation of different proton-ordered ice phases.

## Abstract (web summary)

The preparation of fully proton-ordered ice phases remains challenging and residual entropy usually remains even at the absolute zero. Here, the authors achieve a transition from disordered ice XII to fully ordered ice XIV triggered by HCl doping, which enhances proton transfer dynamics by five orders of magnitude.

## Graphical abstract

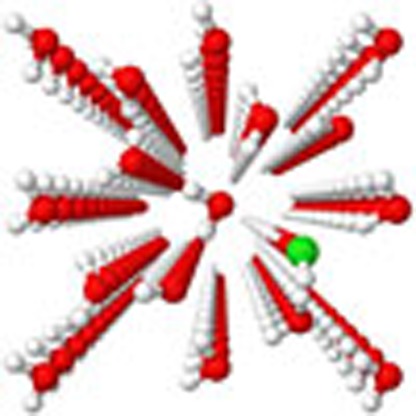

Symmetry breaking order–disorder transitions are ubiquitous and of importance in fields as diverse as physics, chemistry and mathematics. Quite often, the low-temperature ordered phase cannot be accessed because of geometrical frustration and therefore residual entropy remains even at the absolute zero. One of the first examples of residual entropy was pointed out by Pauling to describe water ice^1^. With the exceptions of cubic ice (ice I_c_), ice II, ice IV and ice XVI, all H_2_O ice phases are known to form proton disorder/order pairs, that is, ice I_h_ and XI (with ice I_h_ designating the naturally occurring hexagonal ice), ice III and IX, ice V and XIII, ice VI and XV, ice VII and VIII, as well as ice XII and XIV (refs 2, 3, 4, 5, 6). The degree of proton order on their lattices is accessible using, for example, diffraction and calorimetric techniques. After refinement of diffraction data one obtains fractional occupancies of H atoms in a given unit cell, which represent space/time averages of the configurations sampled during the measurement. This provides information about the degree of order in a static structure, but does not allow one to quantify entropy changes associated with a phase transition. In addition, neutron diffraction requires the use of deuterated samples, which may show a different degree of (dis)order than hydrogenated specimens. By contrast, in calorimetry the latent heat of transformation, Δ*H*, associated with the proton-(dis)ordering transition can be determined directly and the onset temperature, *T*_c_, of latent heat evolution yields the phase equilibrium between ordered and disordered phases. The entropy change, Δ*S=*Δ*H/T*_c_, at the order/disorder transition can be quantified and compared with its theoretical maximum—given (approximately) by the Pauling entropy, Δ*S*_P_=*R* ln(3/2) (refs 1, 7, 8, 9)—which is slightly dependent on the topology of the oxygen network^10^. Pauling’s entropy was found to be of relevance not only in the physics of water ice but also for magnetic spin ice pyrochlores such as Dy_2_Ti_2_O_7_ (ref. 11). However, for many of the ‘ordered’ water ice crystals such as ice IX (refs 12, 13), ice XI (ref. 14) or ice XIV (ref. 3), the reported degree of order is incomplete. Calculations based on the assumption that ice III is fully disordered suggest that ice IX is fully ordered, see ref. 13. However, this assumption is at variance with experimental evidence, see refs 13, 16, 17. Likewise, the degree of disorder is not 100% for the ‘disordered’ forms, except for phases whose space group requires full proton disorder (for example, ice VI and ice XII (ref. 6)). Partial proton order is already present, for example, in the disordered ice phases III and V where, for certain hydrogen bonds, the probability of a proton to be located either on one side or the other displays some fractional value^15^,^16^,^17^. As a consequence, no more than 66% of the Pauling entropy were calorimetrically detected at the ice XIII→V disordering transition^18^ in spite of ice XIII showing only a small degree of residual disorder according to neutron diffraction^2^.

The proton-ordered phases displaying a near-maximum order parameter seem to be the ones most abundantly investigated. Dielectric studies indicate that 83% of the maximum proton order was achieved (91% for deuterated crystals) in ice VIII (ref. 19), whereas neutron diffraction indicates ice VIII to be even completely ordered at ∼2.5 GPa and 10 K^20^,^21^, and ice VII to be completely disordered at 295 K^19^,^22^. The exact nature of the transition between ice VII to VIII and the release of the Pauling entropy at the transition remain unclear. Kuhs *et al.*^20^ measured in the vicinity of the phase transition and concluded that ‘further data on both phases just above and just below the transition temperature will be required to establish higher limits on possible disordering of ice VIII at high temperature and ordering of ice VII at low temperature’. Nelmes *et al.*^23^ concluded that ‘the structure of ice VII is significantly different from previous interpretations and is more complex’. Intermediate phases with frozen-in disorder such as ice VII’ (ref. 24) and evidence for a transition in ice VII involving changes in the character of the proton order/disorder were discussed^25^. Under high-pressure conditions the transition from ice VII to ice VIII proceeds without the aid of dopants or elaborate cooling protocols, because the equilibrium temperature for the proton order–disorder transition at *T*_c_=273 K is rather high. For ices with *T*_c_<150 K, dopants are always required to facilitate proton ordering and thus to prevent immobilization of slow local motions. Order parameters exceeding 82% of the theoretical maximum were apparently never established for ice XI, despite considerable experimental effort^14^. On the one hand, to generate ice XI from ice I_h_, doping with alkali hydroxide, notably KOH, is required^26^,^27^, see also the references cited in chapter 11.2 of ref. 5. Also RbOH-doped ice I_h_ can transform into a proton ordered state, see ref. 26. Particle irradiation may be another option, see ref. 27. On the other hand, HCl is the dopant known to facilitate proton order in ice XIII, XIV and XV (refs 2, 3). Evidently, these dopants generate point ionic and/or Bjerrum (=orientational) defects on the ice lattices and the consequent breaking of the Bernal–Fowler ice rules renders dipolar dynamics such as proton transfer and reorientational water motion possible on them. Thus, doping-mediated unfreezing of the kinetics facilitates the exploration of the system’s ground state. It is plausible, yet largely untested^28^,^29^, that the dynamics in the corresponding proton-disordered precursor phases is enhanced most by that dopant, which facilitates proton order most effectively.

This work shows by means of calorimetry that HCl doping and suitable sample preparation allows one to achieve a transition from completely disordered ice XII to 60% ordered ice XIV, the highest known proton order in ice XIV. Our dielectric spectroscopy studies reveal that the transition is triggered by a five orders of magnitude enhancement of the proton-transfer dynamics induced by HCl doping. The proton-ordering process is shown to be composed of a fast component, yielding about 20% of the Pauling entropy, and a slow component achieving 60% order.

## Results

### Calorimetric entropy determination

**Figure 1:**
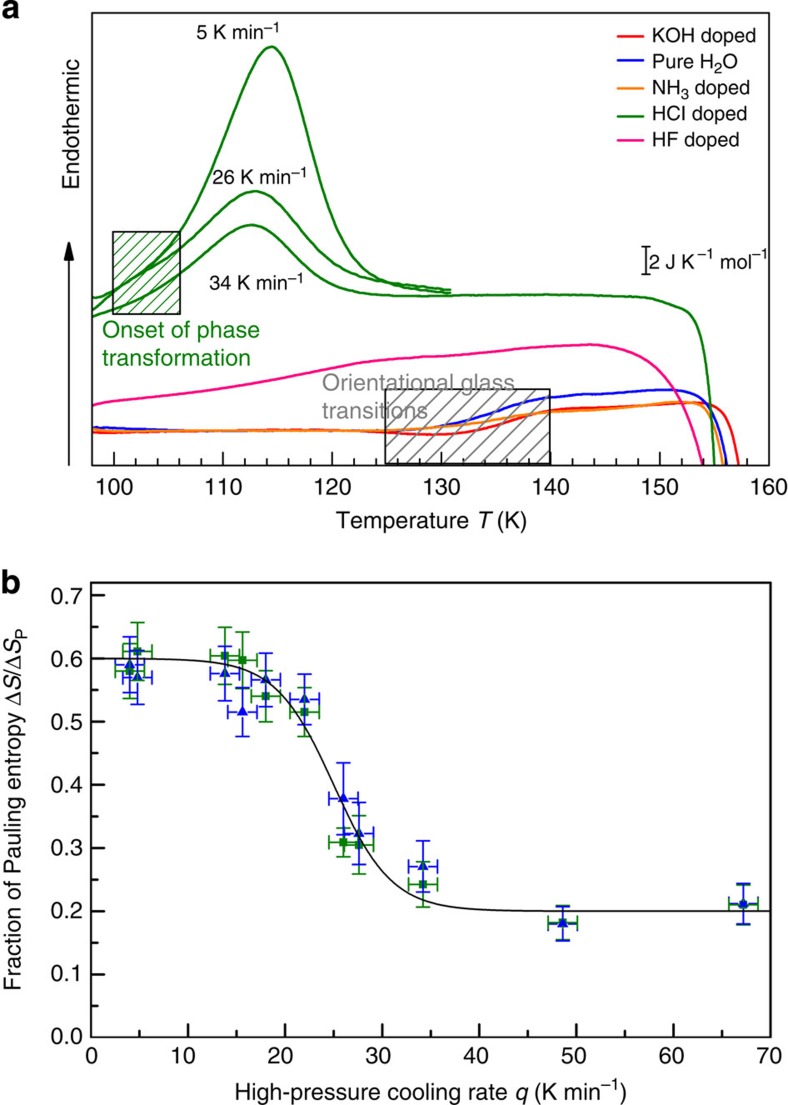
Calorimetric results. (**a**) Ambient pressure heating scans for pure and doped samples. The scans were recorded at 50 K min^−1^ for HCl and HF-doped ice XIV and at 30 K min^−1^ for all ice XII crystals. For HCl-doped samples, the latent heat associated with proton disordering (peak H1, see Methods) increases with decreasing rates, *q*, applied during the prior high-pressure cooling at 0.81 GPa. These rates are given in the figure. Hatched areas indicate the onset of the XIV→XII transition (‘phase transformation’) or the thawing of reorientational mobility in geometrically frustrated ice XII (‘orientational glass transition’). (**b**) Recovered fraction of Pauling entropy in HCl-doped samples as calculated from the peak area ratio H1/H2 (blue triangles) or H1/H3 (green squares) as a function of the high-pressure cooling rate *q* (see Methods). The line is drawn to guide the eye. Each individual point represents an average of typically two or three measurements. The error bars on the *y* axis reflect the reproducibility of Δ*S* and also include ambiguities related to the definition of onset and endpoint used for the peak integration. The error bars on the *x* axis reflect the fluctuation of the cooling rate in the vicinity of and through the transition at 0.81 GPa.

With the goal to establish conditions enabling one to achieve maximum proton order, it is advisable to start from an entirely proton-disordered phase such as ice XII (ref. 30). We cooled ice XII at high pressure (0.81 GPa) from 200 to 77 K with HCl, HF, NH_3_ or KOH as dopants (see Methods). For HCl and HF doping, this results in an ice XII→XIV transition. After recovery of the samples we use dielectric spectroscopy to explore potentially enhanced proton dynamics and calorimetry to examine the degree of order achievable in ice XIV. These experiments are carried out at ambient pressure on heating and they probe the XIV→XII transition, demonstrating that ice XIV can exhibit 60% proton order under suitable high-pressure preparation conditions. Only for HCl-doped ice XIV a pronounced endotherm is detectable at an onset temperature *T*_c_=102±3 K, which indicates conversion from ice XIV to ice XII, see Fig. 1a. This presents the first precise determination of *T*_c_ in protonated ice XIV and is in very good agreement with the onset (near 98 K) of lattice constant changes at the transition of deuterated ice XIV/XII obtained by Salzmann *et al.*^31^ from neutron diffraction. Thus, the isotope effect on the order–disorder transition temperature is small. Figure 1 demonstrates that the transition entropy, Δ*S*, depends on the rate *q* with which HCl-doped ice XII was cooled at 0.81 GPa to temperatures below *T*_c_. For *q*≤15 K min^−1^, we obtain values scattering between about 57% and 63% of Δ*S*_P_=3.4 J K^−1^ mol^−1^, whereas for 25 K min^−1^<*q*<70 K min^−1^ only ∼20% of the Pauling entropy are recovered. This demonstrates that 60% proton order in ice XIV was achieved by cooling HCl-doped ice XII at 0.81 GPa and *q*≤15 K min^−1^ to 80 K. By contrast, for DCl-doped D_2_O ice XIV only partial ordering was found from powder neutron diffraction experiments after cooling the sample at 0.8 K min^−1^ from 180 to 80 K at 1.2 GPa^2^. In the unit cell, two pairs of deuteron atoms were found to show disorder as indicated from fractional occupancies of 0.6/0.4; here, 0.5/0.5 marks full disorder and 1.0/0.0 marks full order (see Table 2 in ref. 2). We interpret this result to indicate that ordering in deuterated ice XII is slower and more difficult to obtain than in protonated ice XII.

For HF doping, only a very weak endotherm can be seen at ∼110–130 K in Fig. 1a, corresponding to ∼6% of the Pauling entropy. Reducing the cooling rate at 0.81 GPa for HF-doped samples by an order of magnitude does not have an effect at all, unlike for the HCl-doped samples. We emphasize that 60% proton ordering can be achieved only under high-pressure conditions, but not at ambient pressure. Even on slow cooling (for example, 5 K min^−1^) in the calorimeter, we regained only ∼12% of the Pauling entropy for the XII→XIV transition (not shown). This finding is consistent with earlier Raman^32^ and neutron diffraction results^31^ that were ‘attributed to the build-up of orthorhombic stress during the tetragonal to orthorhombic phase transition, which the application of pressure may help overcome’^33^. Even the fastest high-pressure cooling rates, *q*>30 K min^−1^, employed in our study resulted in a gain of ∼20% of the Pauling entropy, see Fig. 1b. Doubling the high-pressure cooling rate *q* from ∼34 to 68 K min^−1^ does not reduce the entropy gain. It thus seems that the process of proton ordering in ice XII involves two stages: first, a rapid stage proceeding easily both at ambient and at high-pressure conditions, which yields about 20% of the Pauling entropy, and a second much slower stage that takes place only at high pressures and yields about 40% of the Pauling entropy. It appears plausible that the two processes are related to sets of crystallographically distinct hydrogen atoms, some of which do not order easily. For deuterated ice XIV, these were identified to be the deuteron pairs D4-12 and D5-13 (see Table 2 in ref. 2), which do not order even after very slow high-pressure cooling. For protonated samples, these positions order according to the calorimetric data. This may be related to the annealing effects observed for the entropy changes near the ice I_h_←XI transition in RbOH- and KOH-doped samples^34^.

### Dynamics studied by dielectric spectroscopy

**Figure 2:**
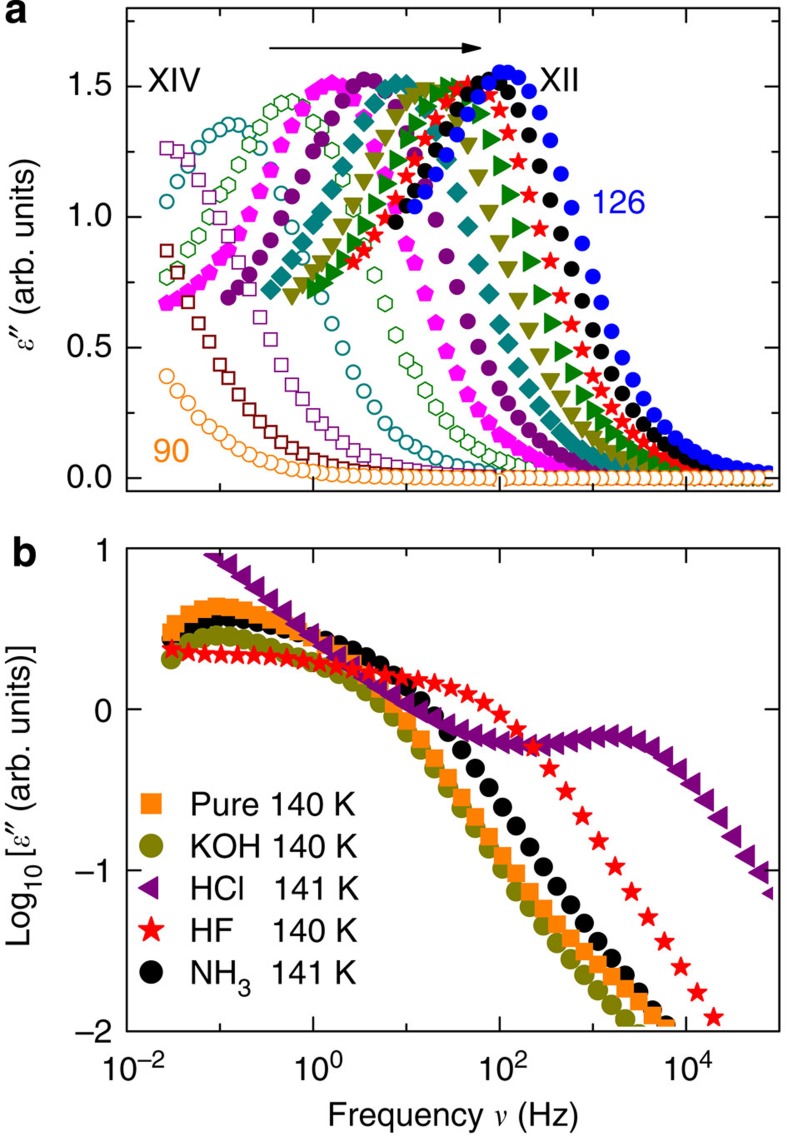
Dielectric loss spectra of ice XII or partially ordered ice XIV. (**a**) Data measured on heating from 90 to 126 K in steps of 3 K for HCl-doped ice XIV (open symbols) and XII (closed symbols). Partial proton ordering in ice XIV reduces its dielectric loss amplitude and hence its static dipolar susceptibility. (**b**) Spectra recorded for doped and undoped ice XII near 140 K. The dynamics in HCl doped samples is evidently the fastest.

**Figure 3:**
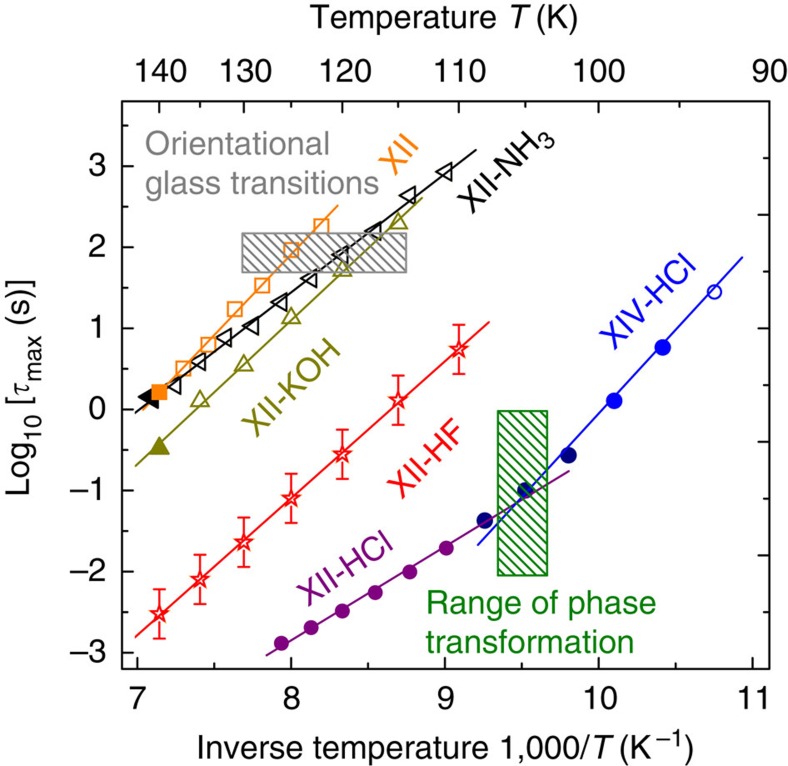
Time scales from dielectric spectroscopy. The data refer either to undoped ice XII or partially ordered ice XIV. Closed symbols refer to resolved peaks; open symbols are derived from frequency temperature scaling. For the HF-doped samples, the uncertainty in the determination of time scales is relatively large. The lower hatched area marks the temperature range in which the order/disorder transition occurs; the upper hatched area highlights the *τ*≈100 s regime, typically associated with (orientational) glass transitions. The solid lines emphasize the considerable difference of the activation energies characterizing the HCl-doped samples, that is, disordered ice XII (*E*=21±2 kJ mol^−1^) and partially ordered ice XIV (*E*=36±3 kJ mol^−1^).

Let us now demonstrate that the ice XIV→XII transition can be resolved not only in thermodynamic but also in dielectric relaxation experiments for samples prepared using a high-pressure cooling rate of *q*=50 K min^−1^. This rate leads to a proton ordering of about 30%, *cf*. Fig. 1b. Dielectric loss spectra, *ε"*(*ν*), recorded every 3 K on heating from 90 to 126 K are presented in Fig. 2a for an HCl-doped crystal. A relaxation peak at *ν*_max_, which defines a motional correlation time *τ*_max_=(2*πν*_max_)^−1^, is seen to shift through the experimentally accessible frequency window. All spectra display essentially the same shape. At the lowest temperatures, the dielectric loss maxima of ice XIV are out of the frequency range. Assuming that similar to that for ice XII, for ice XIV the shape of the loss peak is virtually temperature invariant as well, *cf*. Fig. 2a, frequency temperature scaling yields the time constants marked as open symbols in the Arrhenius diagram, *cf*. Fig. 3. As Fig. 2a shows, the thermal shift of the loss spectra is much stronger in the 90–102 K interval than it is at higher temperatures, signalling the occurrence of the phase transition. In addition, below *T*_c_ the static dielectric relaxation strength drops as expected for (partial) dipolar ordering. Reassuringly, the transition temperature, *T*_c_=104±2 K, resulting from the dielectric experiments, confirms the calorimetrically determined *T*_c_. Near *T*_c_ the activation energy *E* increases by about 70% (from 21 to 36 kJ mol^−1^) as Fig. 3 shows. An even stronger increase of *E* (from 14.6 to 32.6 kJ mol^−1^) was found near the ice I_h_→XI transition^35^.

### Impact of doping

Now, we examine the impact of various doping agents on the potential enhancement of the dipolar dynamics and on the establishment of proton order. To address the latter issue we recorded calorigrams for undoped ice XII as well as for specimens doped with NH_3_ and KOH, *cf*. Fig. 1a: a significant endothermic peak cannot be detected near 102 K, indicating that ice XIV is not formed in any of these samples. Hence, they represent frozen proton-disordered states, the so-called orientational glasses. Such states are already known for ice I_h_, IV, V, VI and undoped XII. Orientational glass transitions were observed for ice I_h_ near 110 K (ref. 36) and for the high-pressure ice phases in the 130–140 K range^37^. An increase in heat capacity at onset temperatures *T*_onset_∼124–130 K in the doped ice XII orientational glasses, *cf*. Fig. 1a, signals motional time scales near 100 s. This indicates that at *T*_c_≈103 K proton mobility is insufficient to achieve the ice XII→ice XIV transition in these crystals.

Dielectric measurements reveal reorientational dynamics also in variously doped ice XII samples. In Fig. 2b we compare their loss spectra with that of the undoped crystal at *T*≈140 K, a temperature at which the loss peaks of all samples are in the accessible frequency window. From Figs 2b and 3, it is evident that the relaxation times in the pure and in the NH_3_- and KOH-doped crystals are four to five decades longer than those in HCl-doped ice XII. Doping with KOH, initially considered a candidate to promote proton ordering in ice V (ref. 38), does enhance the dynamics, but only slightly relative to that of the undoped crystal. Interestingly, in an HF-containing sample, we detect dynamics intermediate between that of pure and HCl-doped ice XII, in accord with the calorimetric observation of its only partial proton ordering.

## Discussion

**Figure 4:**
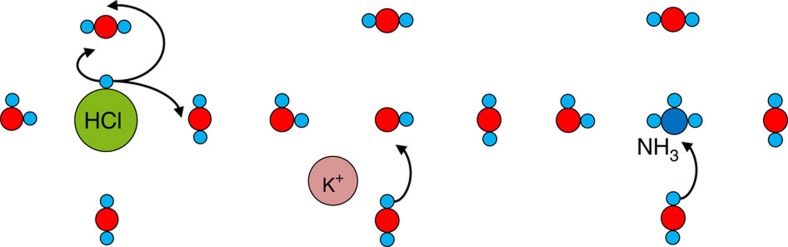
Suggested proton transfers in doped ice. Two-dimensional sketch that illustrates ice-typical defects generated by HCl, KOH or NH_3_ doping. The arrows in this schematic illustration indicate possible proton transfers to the central molecule or ion, or away from it. To keep the illustration compact, the cation/anion pairs are drawn in close spatial proximity while in real ice dissociation is expected.

Thus, together with hints from investigations of ice I_h_ (ref. 29), we conclude that the doping agent enhancing the relaxation dynamics most is indeed the one that ultimately catalyses the establishment of low-temperature proton order. However, how can ices doped by HCl or HF affect the precursor dynamics of the ice XII→XIV transition so differently, *cf*. Fig. 3 (for ice V and XIII see ref. 18)? The answer to this question provides clues regarding the differing efficiencies of the various dopants. The atomic radii for O in KOH and N in NH_3_ are similar, and thus both fit well into the anion site of the ice lattice. By contrast, the Cl^−^ ion radius is about three times larger and will give rise to considerable local lattice distortions. As schematically sketched in Fig. 4, we suggest that this distortion together with the relatively large polarizability of the Cl^−^ ion opens new pathways for proton transfers. In other words, it should be possible that a proton close to a Cl^−^ site can be rotated in any direction compatible with the ice rules. In addition, the F^−^ ion is larger than the O atom, but only by a factor of two, and the expected lattice distortions are not as pronounced. In addition and apart from possible differences in solubility^39^, HF is a weaker acid than HCl. As the effectiveness of HCl was attributed to the creation of H_3_O^+^ defects^39^, weaker acidity reduces the capability to enhance proton ordering in the ice XII lattice significantly, so that HF induces ordering of only a small fraction of protons, see Fig. 1a.

Allowance for additional moves in chlorine’s first coordination shell will result in a sizeable enhancement of the proton motion. In the very compact high-pressure phases such as ice XIII, XIV and XV, or their proton-disordered precursors (with densities *ρ*≥1.23 g cm^−3^ (ref. 40)) lattice distortions can conceivably emanate out to additional shells surrounding a Cl^−^ dopant, thus enhancing the dynamics further. This appears to be different for the ice I_h_ and XI pair (*ρ*≈0.93 g cm^−3^), in which KOH promotes proton ordering, whereas HCl does not. Thus, the role the Cl^−^ ion plays for the high-pressure ices XIII, XIV and XV is obviously taken over by the K^+^ ion (or the larger, yet less efficient Rb^+^ (ref. 26)) for the ice I_h_ lattice, which is distorted by these ions. However, quantitative details regarding the impact of lattice distortions and sterical effects on the local proton transfer and water reorientation rates are largely unclear and call for theoretical work. Our results suggest that these effects might be key in understanding the role of dopants on reorientational defect dynamics.

In summary, we show that NH_3_ and KOH impurities are ineffective in altering the motion in ice XII, whereas for HF doping its speed-up is moderate. Only addition of HCl generates a dramatic dynamic enhancement. In combination with slow cooling at high pressures, this allows us to recover 60% of the Pauling entropy. Our calorimetry data imply that ∼25–35% of all protons can readily be ordered in the presence of HCl dopants, both at high-pressure and at ambient-pressure conditions. The remaining 65–75% of all protons in ice XII can solely be ordered using HCl doping together with slow cooling at high pressure. This suggests that the stresses associated with the change in space group can be released only at high pressures and with a significantly distorted local geometry. Under these conditions, the four to five orders of magnitude enhancement of relaxation dynamics in HCl-doped ice XII that we discovered with dielectric spectroscopy is crucial for establishing the ice XIV phase. Hence, our experimental data show novel routes to an understanding of proton-ordering transitions and thereby paves the way for future computer simulation work directed at ultimately solving the riddle regarding the mechanism driving doping-induced proton-ordering transitions.

## Methods

### Preparation of ice samples

Ice XII can be obtained using different routes^30^,^41^,^42^. In the present work, doped and undoped ice XII was produced by crystallizing high-density amorphous ice (HDA). HDA was prepared according to the original protocol by Mishima *et al.*^43^, that is, by freezing pure water at ambient pressure and then pressurizing hexagonal ice to 1.6 GPa at 77 K. HDA was subsequently decompressed to 0.81 GPa and heated at >25 K min^−1^ to 185 K. At such high heating rates the competing crystallization channel to ice IV is effectively suppressed and pure ice XII forms. This was shown in earlier work for pure water samples^44^ and now it has to be established whether this is also the case for the doped samples used in this study. Doped ice XII was produced by starting from 0.01 M aqueous solutions.

### Characterization of ice XII/XIV samples by powder X-ray diffraction

**Figure 5:**
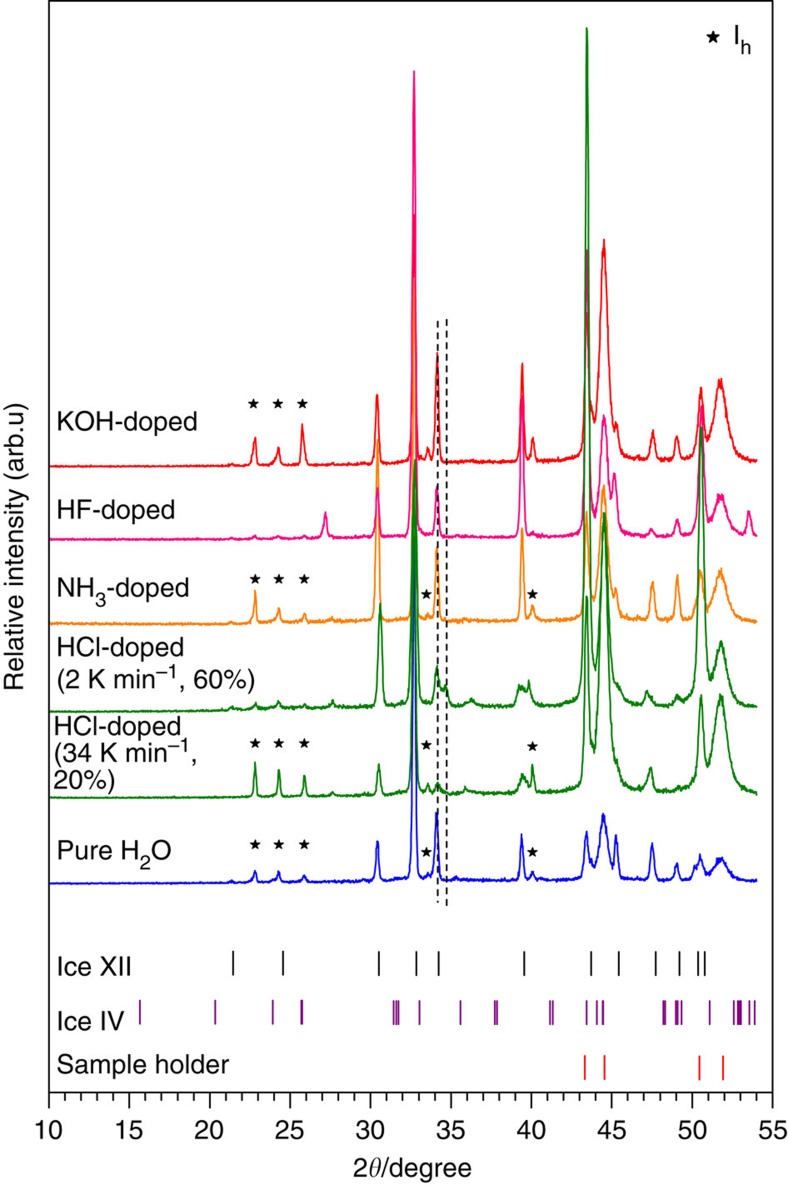
Cu-K_α,1_ powder diffractograms. Pure and doped ice XII/XIV samples were recorded at about 80 K in *θ*–*θ* geometry. In case of HCl doping, two diffractograms are shown, belonging to 60% ordered ice XIV and to 20% ordered ice XIV. Tick marks indicating the most important Bragg reflections for the sample holder (nickel-plated copper), ice IV and ice XII are shown at the bottom. Hexagonal ice condensed on sample transfer on the sample surface is marked by asterisks. The vertical dashed lines mark the ice XIV orthorhombic splitting. Note that the splitting has not yet developed in 20% ordered ice XIV samples. Ice IV contamination is ruled out from these diffractograms.

Preparation of pure ice XII by crystallizing HDA at high-pressure conditions and fast heating rates was established in earlier work^44^. In the present work, the impact of dopants on the proton order/disorder transition was studied. It is conceivable that the dopants not only influence the proton dynamics but also the formation of HDA and its crystallization. For this reason it is necessary to carefully check the nature of all samples after recovering them at 77 K to ambient pressure by X-ray diffraction. The diffractograms shown in Fig. 5 were all recorded at ambient pressure and ∼80 K using a Siemens D5000 diffractometer (Cu-*K*_α,1_, *λ*=1.5406 Å) in *θ*–*θ* geometry. Samples were cold loaded to the sample holder through ambient air, which results in condensation of a tiny amount of hexagonal ice on the sample surface. All powder patterns, both from doped and undoped ices, show the pattern well known for ice XII (refs 30, 41, 42). Apart from traces of hexagonal ice, all diffractograms are free from contamination. In particular, ice IV contamination is not observable in any of the diffractograms, which is known as an alternative crystallization product from HDA at high-pressure conditions. Whereas ice IV crystallizes preferentially at small heating rates, ice XII crystallizes preferentially at large heating rates. None of the Bragg reflections from ice IV are noticeable in Fig. 5. Even the most intense ice IV Bragg reflection at 2*θ*=31.8° does not appear. The contamination of our ice XII samples by ice IV is <1% not only for the undoped crystals but also for all of the doped ice XII specimens studied here. That is, the use of 0.01 M aqueous solutions instead of pure water does not noticeably affect the pressure-induced amorphization of ice I_h_ at 77 K and also does not change the crystallization kinetics of HDA at 0.81 GPa. Even the heating rate dependence of HDA crystallization does not seem to be affected by the introduction of dopants.

Ice XII and ice XIV can be distinguished by X-ray diffraction on the basis of the orthorhombic splitting of the Bragg peak at 2*θ*=34.1° (Cu-*K*_α,1_), see the vertical dashed lines in Fig. 5. We here compare HCl-doped ice XIV samples obtained by slow and fast cooling at high-pressure conditions: The splitting is resolved well for the slowly cooled, 60% ordered sample, but not for the 20% ordered sample (see Fig. 5). This indicates that only the slow component of the ordering process results in the orthorhombic splitting and clarifies that only the slow-ordering component is hindered by orthorhombic stress at ambient pressure. The fast component, resulting in about 20% ordering, by contrast, may take place both at 1 bar and at high-pressure conditions, because it is not afflicted with orthorhombic stress.

### Calculation of fraction of Pauling entropy from calorigrams

**Figure 6:**
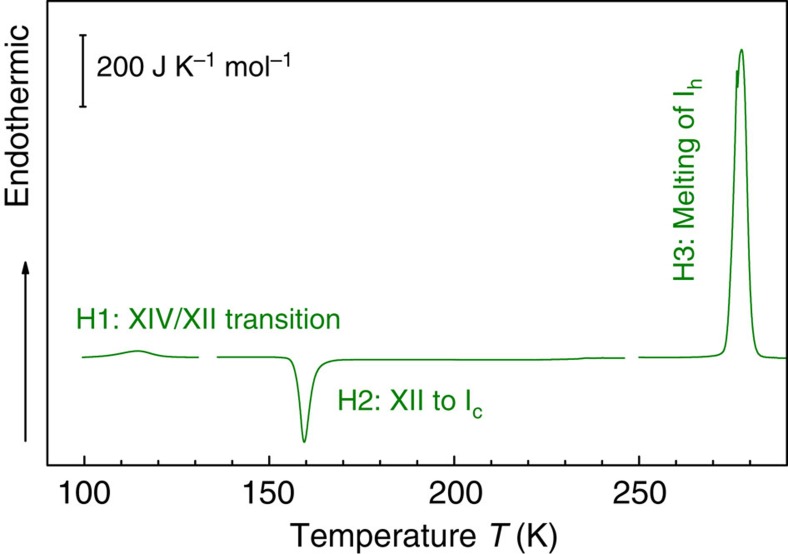
Phase transitions experienced by ice XIV on heating at 1 bar. Peaks in the calorigram indicate latent heats associated with ice XIV→ice XII (endotherm H1), the polymorphic transition ice XII→ice I_c_ (exotherm H2) and ice I_h_ melting (endotherm H3). Peaks H1 and the onset of H2 are shown in a magnified manner in Fig. 1a. The samples were cold loaded at ambient pressure to the instrument. The differential scanning calorimetry protocol involves first heating from 90 to 220 K, converting the sample to ice I_c_ (H1 and H2), cooling back to 90 K and second heating to 300 K, melting the ice sample (H3). The second heating scan is used as a baseline that is subtracted from the first heating scan.

In the present work, three first-order phase transitions associated with latent heat were detected calorimetrically in ordering samples (see Fig. 6). First, the endotherm at *T*_c_≈102 K indicates the proton order–disorder transition from ice XIV to ice XII (see Fig. 1a). The latent heat associated with this transition is called H1 in Fig. 6. Second, the exotherm at *T*_x_≈155 K indicates the polymorphic transition from ice XII to cubic ice. The latent heat associated with this transition is called H2 here and is known from literature to be –1,270±50 J mol^−1^ (ref. 45). Third, the endotherm at *T*_m_=273 K indicates melting of hexagonal ice. This well-studied transformation is called H3 here and is associated with a latent heat of melting of +6,012±30 J mol^−1^ (ref. 46). All onset temperatures for the peaks H1, H2 and H3 are determined by the tangent intersection method. It is noteworthy that the very weakly exothermic transition from cubic ice to hexagonal ice, which takes place in the broad temperature interval at ∼180–230 K cannot be seen at the zoom level used for Fig. 6 and is ignored here.

The transition entropy Δ*S* is extracted from the calorimetry scans either by using the ratio of the peak areas H1/H2 or H1/H3 to determine transition enthalpies, which are then divided by *T*_c_. That is, there are two independent ways of calculating Δ*S* from a single calorigram. The H1/H2 way is shown using blue triangles and the H1/H3 way is shown using green squares in Fig. 1b. For most scans Δ*S* from the two methods differs by <1%, whereas in some scans the difference goes up to 10%, see Fig. 1b. Therefore, both evaluation methods are shown in Fig. 1b separately. A couple of scans were identified as outliers and discarded from the data set. The unreasonably low values of these outliers indicate that part of the H1 peak is missing, because the sample has partly transformed outside the instrument by inadvertent heating. Please note that the error bars are smaller for small Δ*S* values, because data points in Fig. 1b are depicted as Δ*S/*Δ*S*_P_, that is, divided by the Pauling entropy. The relatively large scatter is expected, because in the past individual latent heat (that is, H2) measurements were seen to fluctuate by up to ±10% (refs 42, 45) and the data in Fig. 1b are calculated from the ratio of two latent heats.

### Dielectric spectroscopy

The dielectric response of the ice crystals was measured using an Alpha-A impedance analyser as detailed earlier^47^. To this end, the crystal powder was cold loaded at ambient pressure into a parallel plate capacitor of a Quatro cryosystem and care was taken that the sample temperature remained below 90 K. The exact filling factor of the cell was not determined and therefore an arbitrary units scale is used for the dielectric loss spectra reported above. The temperature stability during each frequency scan was better than ±0.1 K.

As described in the main text, the relaxation times could be determined in a straightforward manner from the dielectric loss peaks for all samples, except for those doped with HF. Here, the electrical conductivity was larger than that of all other ice XII specimens. Time scales for the HF-doped samples were estimated from approximate shift factors relative to the loss curves of the other crystals. During the preparation, the HF-doped samples, unlike all others, were found to be sticky, that is, electrostatically charged, presumably causing the enhanced conductivity.

## Additional information

**How to cite this article:** Köster, K. W. *et al.* Dynamics enhanced by HCl doping triggers 60% Pauling entropy release at the ice XII–XIV transition. *Nat. Commun.* 6:7349 doi: 10.1038/ncomms8349 (2015).
