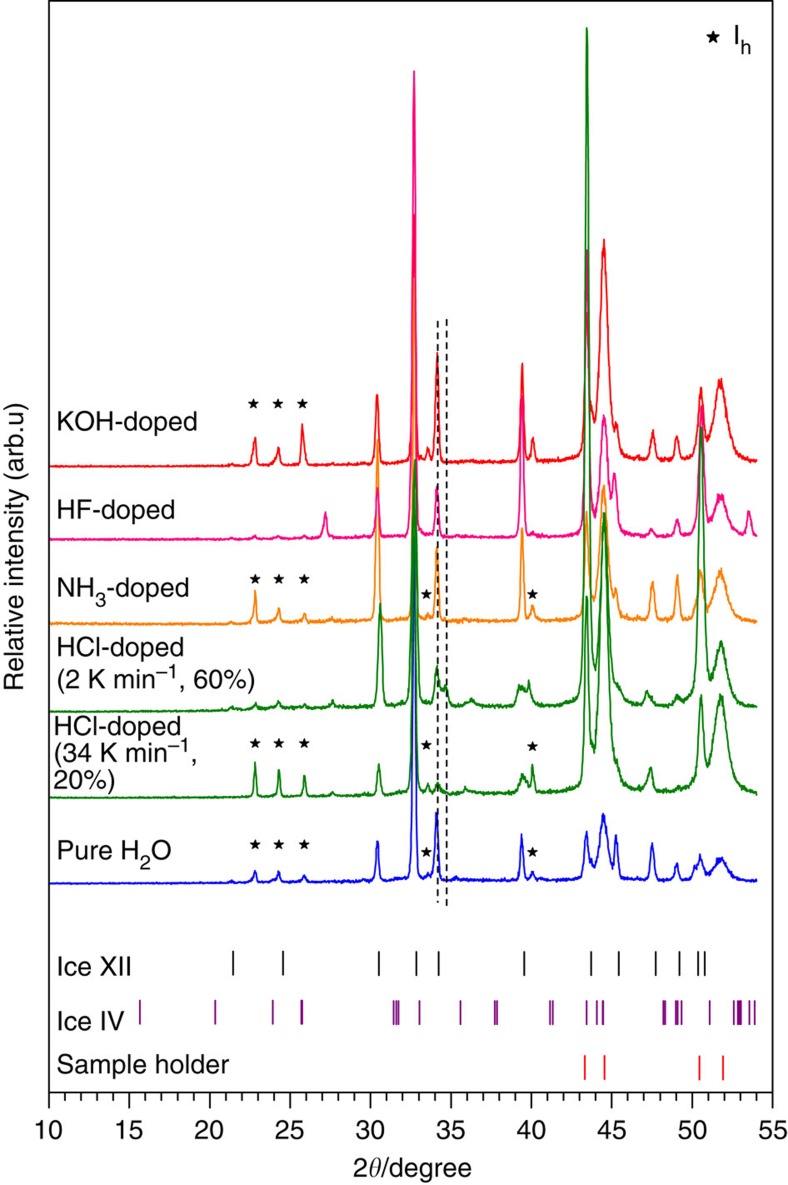# Author Correction: Dynamics enhanced by HCl doping triggers 60% Pauling entropy release at the ice XII–XIV transition

**DOI:** 10.1038/ncomms16189

**Published:** 2018-06-20

**Authors:** K. W. Köster, V. Fuentes-Landete, A. Raidt, M. Seidl, C. Gainaru, T. Loerting, R. Böhmer

Nature Communications 6: Article number: 7349 ; DOI: 10.1038/ncomms8349 (2015); Published online 06 16 2015; Updated 06 20 2018

We became aware of a mistake related to the integration of peaks observed in our calorimetry experiments. In particular, the peak area is proportional to the heating rate. In our data analysis this proportionality factor was accidentally considered twice, namely once by the Pyris software controlling our calorimeter (Perkin Elmer, DSC8000) and once by ourselves. The recovered fraction of Pauling entropy reported is calculated from the area ratio of one endotherm (H1, ice XIV to ice XII transition) and one exotherm (H2, ice XII to ice I transition) or a second endotherm (H3, melting of ice). We typically recorded H1 at 50 K/min and H2 and H3 at 30 K/min. As a consequence the fraction of Pauling entropy was erroneously overestimated by a factor of 50/30. In order to correct this mistake the data in Fig. 1b need to be multiplied by 30/50=0.6. The only exception is the point recorded for the high-pressure cooling rate of 33 K/min, for which both heating rates were 30 K/min—i.e., it does not need to be corrected. As a consequence, our claim for "full Pauling entropy release" and "complete proton order in ice XIV" cannot be sustained.

As a result of this, the following changes have been made to the originally published version of this Article:

The original title “Dynamics enhanced by HCl doping triggers full Pauling entropy release at the ice XII–XIV transition” was incorrect. The corrected version replaces the word “full” with “60%”.

The fourth sentence of the abstract originally read “Here we report calorimetric measurements on suitably high-pressure-treated, hydrogen chloride-doped ice XIV that demonstrate just this at the transition to ice XII.” The phrase “at the maximum 60% of the Pauling entropy is released” now replaces “just this”.

The first sentence of the last paragraph of the Introduction originally read “This work shows by means of calorimetry that HCl doping and suitable sample preparation allows one to achieve a transition from completely disordered ice XII to fully ordered ice XIV.” In the corrected version, “fully” is replaced by “60%”.

The last sentence of the last paragraph of the Introduction originally read “The proton-ordering process is shown to be composed of a fast component, yielding ∼30% of the Pauling entropy, and a slow component achieving full order.” In the corrected version, “30%” is replaced by “20%”, and “full” is replaced by “60%”.

The fifth sentence of the “Calorimetric entropy determination” section of the Results originally read “These experiments are carried out at ambient pressure on heating and they probe the XIV→XII transition, demonstrating that ice XIV can exhibit complete proton order under suitable high-pressure preparation conditions.” In the corrected version, “complete” is replaced by “60%”.

The tenth and eleventh sentence of the “Calorimetric entropy determination” section of the Results originally read “For *q*≤15 K min^−1^, we obtain values scattering between about 95% and 105% of Δ*S*_P_=3.4 J K^−1^ mol^−1^, whereas for 25 K min^−1^<*q*<70 K min^−1^ only ∼25%–35% of the Pauling entropy are recovered. This demonstrates that complete proton order in ice XIV was achieved by cooling HCl-doped ice XII at 0.81 GPa and *q*≤15 K min^−1^to 80 K.” In the corrected version, “95% and 105%” is replaced by “57% and 63%”, “25%–35%” is replaced by “∼20%” and “complete” is replaced by “60%”.

The last sentence of the first paragraph of the “Calorimetric entropy determination” section of the Results originally read “Thus, the results reported here represent the first example in which the full Pauling entropy could be recovered, demonstrating a transition from fully disordered ice XII to fully ordered ice XIV.” In the corrected version, this sentence has been removed.

The first sentence of the second paragraph of the “Calorimetric entropy determination” section of the Results originally read “For HF doping, only a very weak endotherm can be seen at ∼110–130 K in Fig. 1a, corresponding to ∼10% of the Pauling entropy” In the corrected version, “10%” is replaced by “6%”.

The third and fourth sentence of the second paragraph of the “Calorimetric entropy determination” section of the Results originally read “We emphasize that complete proton ordering can be achieved only under high-pressure conditions, but not at ambient pressure. Even on slow cooling (for example, 5 K min^−1^) in the calorimeter, we regained only ∼20% of the Pauling entropy for the XII→XIV transition (not shown).” In the corrected version, “complete” is replaced by “60%”, and “20%” is replaced by “12%”.

The sixth sentence of the second paragraph of the “Calorimetric entropy determination” section of the Results originally read “Even the fastest high-pressure cooling rates, *q*>30 K min^−1^, employed in our study resulted in a gain of ∼25%–35% of the Pauling entropy, see Fig. 1b” In the corrected version, “25%–35%” is replaced by “20%”.

The eighth sentence of the second paragraph of the “Calorimetric entropy determination” section of the Results originally read “It thus seems that the process of proton ordering in ice XII involves two stages: first, a rapid stage proceeding easily both at ambient and at high-pressure conditions, which yields about 25%–35% of the Pauling entropy, and a second much slower stage that takes place only at high pressures and yields about 65%–75% of the Pauling entropy.” In the corrected version, “25%–35%” is replaced by “20%”, and “65%–75%” is replaced by “40%”.

The second sentence of the second paragraph of the “Characterization of ice XII/XIV samples by powder X-ray diffraction” section of the Methods originally read “We here compare HCl-doped ice XIV samples obtained by slow and fast cooling at high-pressure conditions: The splitting is resolved well for the slowly cooled, 100% ordered sample, but not for the 30% ordered sample (see Fig. 5).” In the corrected version, “100%” is replaced by “60%”, and “30%” is replaced by “20%”.

The last sentence of the second paragraph of the “Characterization of ice XII/XIV samples by powder X-ray diffraction” section of the Methods originally read “The fast component, resulting in about 30% ordering, by contrast, may take place both at 1 bar and at high-pressure conditions, because it is not afflicted with orthorhombic stress.” In the corrected version, “30%” is replaced by “20%”.

The third sentence of the third paragraph of the ‘Discussion’ section originally read “In combination with slow cooling at high pressures, this allows us to observe a transition from fully proton ordered to fully proton disordered ice and thus to recover the complete Pauling entropy.” In the corrected version, “this allows us to observe a transition from fully proton ordered to fully proton disordered ice and thus to recover the complete Pauling entropy” is replaced by “this allows us to recover 60% of the Pauling entropy.”

The second sentence of the caption to Fig. 5 originally read “In case of HCl doping, two diffractograms are shown, belonging to fully ordered ice XIV and to 30% ordered ice XIV.” In the corrected version, “fully” is replaced by “60%”, and “30%” is replaced by “20%”.

The sixth sentence of the caption to Fig. 5 originally read “Note that the splitting has not yet developed in 30% ordered ice XIV samples.” In the corrected version, “30%” is replaced by “20%”.

In the original version of Fig. 1b, the *y*-axis scale was wrong by a factor of 0.6, except for the points at *q*=33 K min^–1^. The correct version of Fig. 1b appearing below as Figure 1:

replaces the previous incorrect one, which appears below as Figure 2:

In the original version of Fig. 5, the label on the fourth curve from the top was incorrectly given as “HCl doped (2 K min^-1^, 100%)” instead of the correct “HCl doped (2 K min^-1^, 60%)”. Similarly, the label on the fifth curve was incorrectly given as “HCl doped (34 K min^-1^, 30%)”, instead of the correct “HCl doped (34 K min^-1^, 20%)”.

The correct version of Fig. 5 appears below as Figure 3:

These errors have now been corrected in both the PDF and the HTML versions of the Article. Except for the entropies deduced from the calorigrams all statements reported remain as they stand.

The authors acknowledge Christoph G. Salzmann (University College London) for calling our attention to these issues.

## Figures and Tables

**Figure 1:**
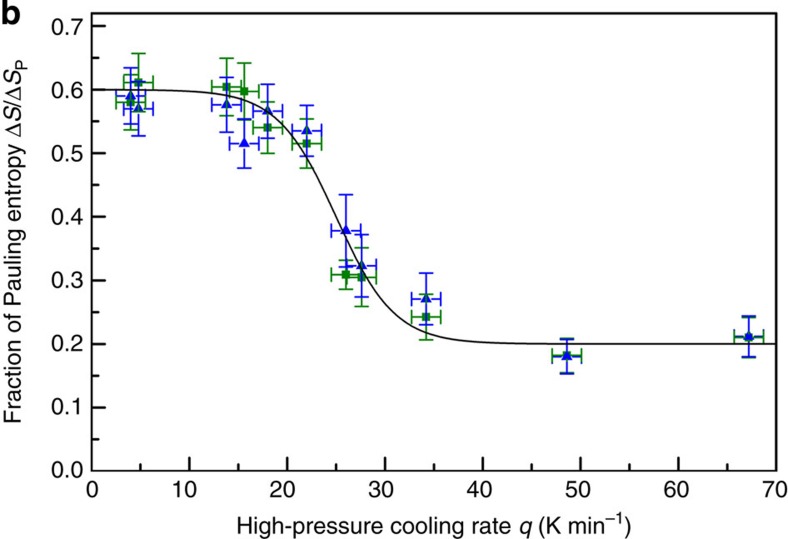


**Figure 2:**
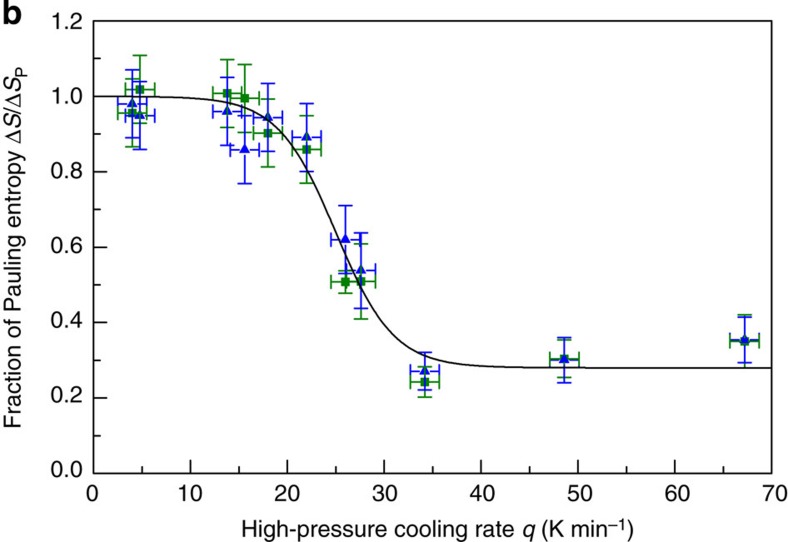


**Figure 3:**